# CEGA—a catalog of conserved elements from genomic alignments

**DOI:** 10.1093/nar/gkv1163

**Published:** 2015-11-02

**Authors:** Aline Dousse, Thomas Junier, Evgeny M. Zdobnov

**Affiliations:** Department of Genetic Medicine and Development, University of Geneva Medical School, Swiss Institute of Bioinformatics, rue Michel-Servet 1, 1211 Geneva, Switzerland

## Abstract

By identifying genomic sequence regions conserved among several species, comparative genomics offers opportunities to discover putatively functional elements without any prior knowledge of what these functions might be. Comparative analyses across mammals estimated 4–5% of the human genome to be functionally constrained, a much larger fraction than the 1–2% occupied by annotated protein-coding or RNA genes. Such functionally constrained yet unannotated regions have been referred to as conserved non-coding sequences (CNCs) or ultra-conserved elements (UCEs), which remain largely uncharacterized but probably form a highly heterogeneous group of elements including enhancers, promoters, motifs, and others. To facilitate the study of such CNCs/UCEs, we present our resource of Conserved Elements from Genomic Alignments (CEGA), accessible from http://cega.ezlab.org. Harnessing the power of multiple species comparisons to detect genomic elements under purifying selection, CEGA provides a comprehensive set of CNCs identified at different radiations along the vertebrate lineage. Evolutionary constraint is identified using threshold-free phylogenetic modeling of unbiased and sensitive global alignments of genomic synteny blocks identified using protein orthology. We identified CNCs independently for five vertebrate clades, each referring to a different last common ancestor and therefore to an overlapping but varying set of CNCs with 24 488 in vertebrates, 241 575 in amniotes, 709 743 in Eutheria, 642 701 in Boreoeutheria and 612 364 in Euarchontoglires, spanning from 6 Mbp in vertebrates to 119 Mbp in Euarchontoglires. The dynamic CEGA web interface displays alignments, genomic locations, as well as biologically relevant data to help prioritize and select CNCs of interest for further functional investigations.

## INTRODUCTION

Genome sequencing provides access to the complete repertoire of inherited functional elements, from encoded genes to regulatory sequences, but recognizing these elements and understanding their biological activities remains challenging. Comparative genomics offers an approach to help recognize such elements, by identifying sequences that remain conserved across multiple species over millions of years of evolution (1). Their intolerance to mutations, making them appear as conserved, implies functional constraints on such sequences, regardless of our knowledge of their functions. Applying such methods to the increasing number of sequenced genomes has helped to identify core genes conserved across many species and has additionally revealed a repertoire of genomic elements at least as large as that of protein-coding genes that does not encode proteins or RNA genes (2–5). These elements were termed Conserved Non-Coding sequences (CNCs) (6,7), and the investigation of their functional roles is still ongoing. Some of the most highly-conserved elements in vertebrates, termed Ultra-Conserved Elements (UCEs) have been tested *in vivo*, but only about half of these showed any capacity for specific *cis-*regulatory activities (8).

Beyond the lack of systematic experimental investigations of such CNCs or UCEs, the variable technical definitions used to classify such elements have hampered progress in this field of research. For example, the working definition of CNCs from pioneering studies (9) selected an arbitrary threshold of a minimum sequence identity over a minimum alignment length in pairwise sequence comparisons, which is still a frequently used definition. However, with no systematic approach to select threshold parameters, the results from employing such a definition are clearly impacted by the evolutionary distance between the pair of species being compared. Various strategies have been developed to fine-tune these definitions in order to generate genome-scale resources of computationally-identified CNCs and to help prioritize candidates in turn satisfying the growing interest in developing functional screens of these elements. Most of the existing resources employ pairwise DNA alignments as a starting point to define CNCs, e.g. human and mouse (VISTA enhancer browser (8)), human and chicken (UCNEbase (10)), human and zebrafish (cneViewer, (11)), or human and fugu (CONDOR (12)). However, pairwise alignments lack comparative power, and ignore the additional evolutionary information to be gleaned from including any of the dozens of vertebrate genomes already sequenced. Extending such approaches by searching ‘seed’ CNCs identified from pairwise comparisons to other species does not completely resolve this issue. If a conserved element is not present (or sequenced) in the species chosen for the initial pairwise comparison, it will not be part of the final set of CNCs. Thus, pairwise approaches are inherently biased and not comprehensive. Similarly, the extension of the pairwise approach to several species, by the choice of a reference organism and subsequent alignments to it, is also biased and not very sensitive to distantly-related species. In addition, definitions of conservation vary considerably, e.g. 100% identity over ≥200 bp for the VISTA enhancer browser, ≥95% identity over ≥200 bp for UCNEbase, user-defined conservation, length, or distance cutoffs for cneViewer, 70–100% identity over ≥30 bp or ≥50 bp for ANCORA (13) (depending on the species pair being considered), ≥70% identity over ≥100 bp (human–mouse) and ≥65% identity over ≥50 bp (mammal–fugu) for TFCONES, ≥65% identity over ≥40 bp for CONDOR and 100% identity over ≥200 bp in human, mouse and rat for UCbase2.0 (14). Some of these resources additionally provide access to the results of regulatory screening of CNCs. Notably, the Vista enhancer browser indexes the results of gene enhancer activity in transgenic mice for 2192 elements, and around a hundred of the 7000 CNCs in CONDOR were tested *in-vivo* for enhancer activity in zebrafish embryos.

To advance the field of research on the identification and characterization of CNCs, we devised a computational pipeline to yield a comprehensive and unbiased set of conserved elements at different radiations along the vertebrate lineage. We overcome the limitations of pairwise approaches by harnessing the power of multiple species comparisons to detect genomic elements under purifying selection using at least five species (15). We gain sensitivity for identifying CNCs by employing global sequence alignments without a reference organism (16) for each of the collinear genomic blocks defined by protein orthology. We rely on phastCons (17) phylogenetic modeling to objectively define conserved elements. The resulting catalog of Conserved Elements from Genomic Alignments (CEGA) provides access to these sets of conserved elements from http://cega.ezlab.org, 24 488 CNCs in the vertebrate clade to 612 364 CNCs in the euarchontoglires (Supraprimates). The CEGA web interface allows browsing of all conserved elements annotated as coding, intergenic or intronic with complementary features selected from the The Encyclopedia of DNA Elements (ENCODE) data (18) such as chromatin state annotations (19) which provide clues to their possible biological functions.

## IDENTIFICATION OF CONSERVED GENOMIC ELEMENTS

**Figure 1.**
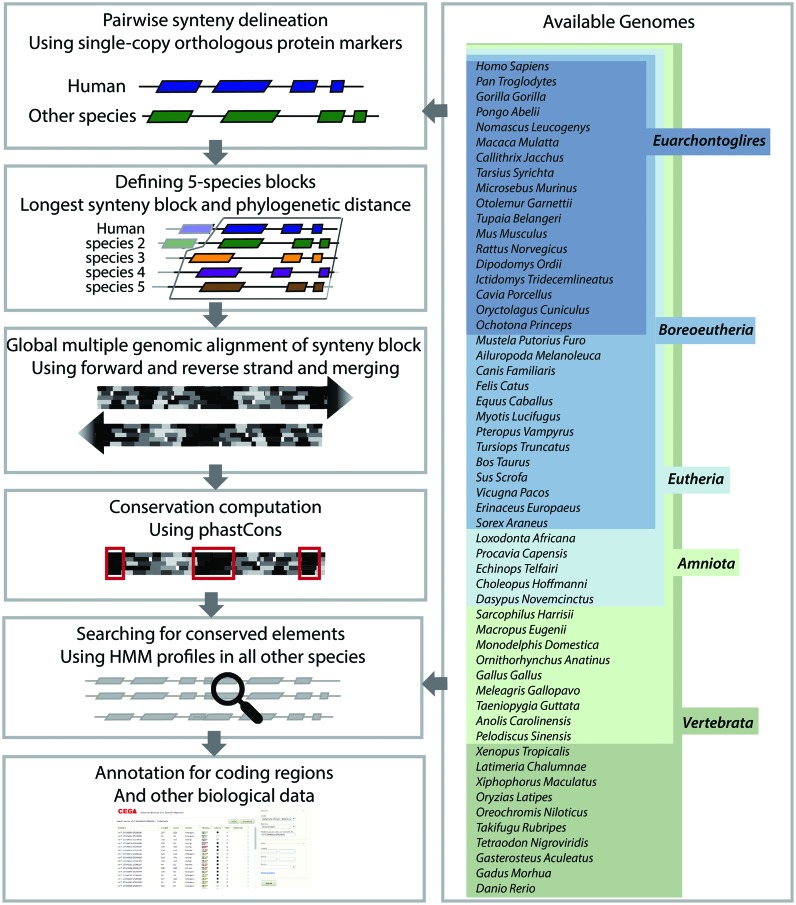
Workflow of CEGA identification of conserved elements.

The CEGA resource presents sets of conserved genomic elements computed independently from a total of 55 vertebrate species. Conservation of a genomic element in a set of species implicitly refers to its presence in the last common ancestor (LCA) of these species. We therefore independently considered five different vertebrate clades: vertebrates, amniotes, Eutheria, Boreoeutheria and Euarchontoglires, each referring to a different LCA and thus to an overlapping but varying set of CNCs. With a total of 1 398 498 CNCs, CEGA offers a comprehensive catalog of conserved elements at each level of the vertebrate phylogeny (Table 1), a third of which are intergenic and the remaining two thirds are intronic. The steps that comprise CEGAs computational pipeline to identify CNCs are explained below and in further details in the Supplementary Material as well as schematically outlined in Figure 1.

**Table 1.** CEGA data content

	Vertebrata	Amniota	Eutheria	Boreoeutheria	Euarchontoglires
Input species	55	45	36	31	18
Included species	43	42	36	31	18
Synteny blocks	1649	1880	1713	1319	1326
Synteny block length^a^ [Mb]	607	1479	1677	1763	1830
Conserved elements	66 280	361 876	869 050	801 032	742 702
CNCs	24 488	241 575	709 743	642 701	612 364
Median CNCs length [bp]	190	147	108	116	128
Total CNCs length [Mb]	6	52	122	116	119

^a^Total synteny block length across the human genome.

### Synteny block delineation

The CEGA pipeline starts with the identification of collinear genomic blocks, also termed synteny blocks, to then be able to perform reliable alignments of the sequences from each block. Delineation of these blocks is based on single-copy orthologous protein markers from OrthoDB (release7) (20). Protein-based markers provide the advantages of having a slower rate of sequence evolution, additional informational content at the amino acid level, and longer sequences than DNA markers used by other approaches to identify orthologous relations and synteny blocks, e.g. in Enredo (21). After looking for synteny blocks between pairs of species, CEGA defines blocks with sets of five species, sufficient to harness the comparative power (15). To maximize the coverage of synteny blocks across the human genome and to take advantage of the growing number of available vertebrate genomes, each block may be defined by different sets of species. In practice, we developed a scoring system based on the phylogenetic distance between each pair of species, the length of the block in terms of number of orthologous protein markers, and the genome sequence quality in terms of gaps in the assemblies to automatically select the best combination of species. Species selection is constrained to contain human and at least one organism from the root level (i.e. most distant from human) of the investigated clade in order to fully span the phylogeny. The common markers identified in all five pairwise blocks across are extracted and the corresponding genomic sequence is further extended by 15 Kb flanks in each genome to include additional intergenic sequences. Employing this strategy resulted in large fractions (from 608 Mb to 1’830 Mb) of the human genome being delineated in to synteny blocks (Table 1).

**Figure 2.**
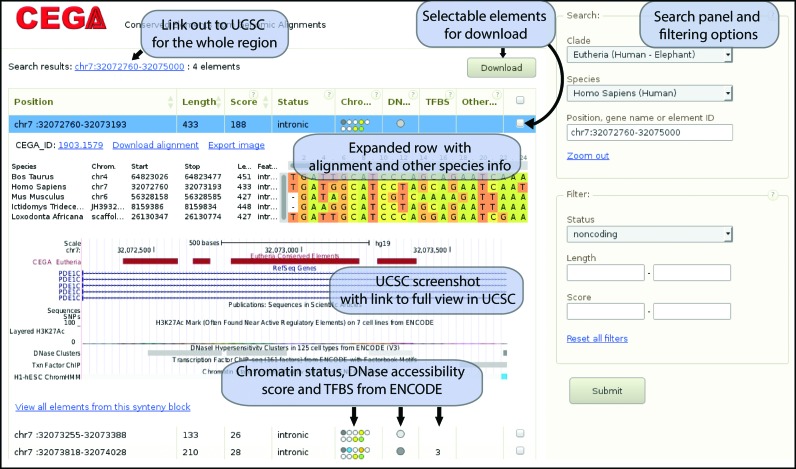
CEGA user interface.

### Multiple sequence alignments

Although we focused on human, requiring all synteny blocks to contain human genomic sequence, CEGA aims to provide unbiased alignments, without using a reference organism. We also target conservation across large evolutionary distances (human – fish) requiring sensitive alignments. Since the protein-orthology-based identification of synteny blocks implies the orthology of the corresponding genomic regions, we opted to use global alignment approaches that attempt to align sequences along the whole lengths of the genomic sequences. After extensive benchmarking of many available alignment methods, we selected MLAGAN (16) that provides global alignments with a local anchoring strategy without requiring the selection of a reference species. To overcome the ‘Heads or Tails’ bias problem (22), i.e. obtaining different results when aligning the exact same sequences in the forward and reverse orientations, we aligned each of the five sequences of the synteny blocks both on the forward and the reverse strands and reconciled the alignments by merging them with MergeAlign (23).

### Identification of conservation

To avoid selecting arbitrary identity and lengths thresholds, the classification of CEGA sets of conserved element employs phylogenetic modeling with phastCons (17) to define evolutionarily constrained elements. The conservation metrics reported are log-odds scores of the probability of the element following a conserved model rather than an unconserved model (parameters and models are described in detail in the supplementary material). Finally, elements with ‘N’ sequence stretches or having less than 20 nucleotides aligned were filtered out of the conserved set.

### Expanding to other species

Since conserved elements are initially identified from a subset of only five species, we used a hidden Markov model (HMM) profile built with *nhmmer* from HMMER 3.1 (24) using each individual element to search whole genome assemblies of all other species. These searches were carried out using the set of vertebrate elements to search all vertebrate genomes. A similar strategy was used for the amniote clade, but limiting the searches to those synteny blocks with previously identified orthologous regions in other species. The highest scoring significant match (*e*-value <0.05) was selected and the alignment of the element recomputed using muscle (25). Currently, CEGA only presents HMM-expanded elements for Vertebrate and Amniotes. In further development the same procedure can be applied to the other clades.

### Functional annotation

Based on Ensembl gene annotations (26) for all species, elements were annotated as either protein-coding, RNA-coding (micro-RNA and long non-coding RNA), intronic or intergenic. These classifications are complemented with selected annotation from the ENCODE project (18) were incorporated such as the chromatin state (19), the number of transcription factors that bind to the genomic region (27), and DNase accessibility values (28). These represent informative annotations that offer additional evidence to help select elements for future investigations of their biological function investigations, e.g. *cis*-regulatory activities. In addition, overlapping ultra-conserved elements defined by alternative approaches available from other databases are listed for easy cross-referencing.

## CEGA DATABASE CONTENT

CEGA database is structured into five main data tables, for each of the investigated vertebrate clade: Vertebrates, Amniotes, Eutheria, Boreoeutheria and Euarchontoglires. In each table, conserved elements are organized into synteny blocks, and then per element. Each element has an ID and information about its location in each species, as well as its sequence and the corresponding annotations. The synteny blocks cover from 20% to 63% of the human genome, with CNCs ranging from 1% to 6.5% of these blocks, depending on the level of the vertebrate phylogeny.

### CEGA web interface

The database is accessible through a dynamic web interface browsable by selecting a region of interest on a human chromosome view or by submitting a genomic location or a gene of interest. In the latter case, the gene genomic position is retrieved from annotations (26) and further expanded with 1 Mbp flanks. The main CEGA display is a table showing the previously described information about each conserved element overlapping the submitted locus. Each row is expandable by just one click to view the sequence alignment from the element, its details in the other species and a screenshot of the genomic location of the element from the UCSC genome browser (29). Access to UCSC browser displaying CEGA tracks can be made directly from the whole region table or from the element view. This functionality is intended as an entry point for the analysis of further biologically-relevant annotations.

As shown on the example of CEGA interface on Figure 2, three columns of the table represent selected biological data from ENCODE that can help to make a selection of relevant elements. The DNAse column shows the DNase sensitivity of the locus with a grayscale, from white for no data or 0 scoring to black for the highest score. This score is based on the combination of the DNAse sensitivity in 125 cell-types (28). Regulatory regions are usually DNase sensitive. The regulatory potential of the element is further detailed by the chromatin state column. Nine circles, one for each of the investigated cell lines, are colored according to the type of activity the integration of chromatin marks data (19) suggests for the genomic region; warm colors represent promoter and enhancer regions whereas cold ones suggest repressed or repetitive region and heterochromatin. The TFBS column simply shows the number of transcription factors with a ChipSeq peak overlapping the conserved element in any of the tested cell lines. This number allows for the selection of highly interacting element over others. A last column is dedicated to the overlap of each CEGA element with ultra-conserved elements identified by other methods. These databases provide other information: gene regulatory blocks and potential gene regulated in UCNE and experimental annotations in CONDOR and Vista enhancer browser.

A checkbox allows the user to select its elements of interest and get bed or Fasta files for them. Bed files can be used as UCSC tracks, looking for overlaps with specific markers and Fasta is provided to look for similar elements or to explore their evolutionary history. The complete set of CEGA data is also available for bulk download.

## CONCLUSIONS AND PERSPECTIVES

CEGA aims to provide an easy access to unbiased and comprehensive sets of CNCs at distinct levels of the vertebrate lineage. The sets were computed based on a strategy to be as comprehensive and sensitive as possible, while keeping scalability in mind. The strategy of using five species per block can cope with the rapidly increasing number of sequenced genomes while harnessing the comparative power. In the future more species can be included without becoming a computational hurdle. This method has however a drawback of not presenting a constant collection of species, not all conserved elements were computed in all species. CEGA provides a convenient access using dynamic webpages to all elements within a genomic interval or close to a particular gene. Quick visualization of relevant biological data in relation to the conserved elements is also provided and can help prioritize the in-depth investigation of a sub-group of elements. Therefore elements can be selected and downloaded in various formats: as bed-file for visualization and for finding overlaps with other features, as multiple alignments in Fasta format for phylogenetic studies or single sequence Fasta for further studies and comparisons.
